# Sympathovagal Imbalance in Drug-Naïve Chronic Obstructive Pulmonary Disease Patients: A Physiological Mechanism to Cope With the Severity of Airway Obstruction in an Observational Study

**DOI:** 10.7759/cureus.100111

**Published:** 2025-12-26

**Authors:** Durgesh K Gupta, Shibu S Awasthi, Suman Gupta, Himani H More

**Affiliations:** 1 Physiology, Dr. KNS Memorial Institute of Medical Sciences (MIMS), Barabanki, IND; 2 Biochemistry, Hind Institute of Medical Sciences, Lucknow, IND; 3 Physiology, Autonomous State Medical College (ASMC), Amethi, IND

**Keywords:** copd: chronic obstructive pulmonary disease, fev1 (forced expiratory volume in one second), fev1/fvc%, forced vital capacity (fvc), heart rate variability (hrv), low-frequency/high-frequency ratio (lf/hf ratio)

## Abstract

Introduction: In the modern world, chronic obstructive pulmonary disease (COPD) is known to affect a large subset of the middle-aged and elderly adult population, primarily due to the increasing prevalence of smoking addiction, indoor air pollution, and poor air quality index in metropolitan cities. We hypothesize that, in an attempt to cope with this respiratory distress, the body reacts with an overdriven sympathetic discharge, which helps maintain pulmonary airway patency and causes sympathovagal imbalance. Heart rate variability (HRV) is a neurophysiological test used to assess sympathovagal balance. A higher HRV signifies normal parasympathetic dominance. The low-frequency-to-high-frequency (LF/HF) ratio is a parameter of HRV that is directly correlated with sympathovagal balance and inversely correlated with HRV. The objective of our study was to compare the sympathovagal balance between COPD patients (cases) and healthy subjects (controls) and find a correlation between sympathovagal balance and spirometry parameters in both cases and controls.

Methodology: A group of 72 drug-naïve COPD patients (diagnosed according to GOLD criteria) were included as cases, and 72 age- and sex-matched, apparently healthy individuals were enrolled as controls in this case-control study. Their spirometry was performed, and the percentage of expected forced expiratory volume in the first second (% FEV1), percentage of expected forced vital capacity (% FVC), and percentage of expected forced expiratory volume in the first second-to-forced vital capacity ratio (% FEV1/FVC ratio) were determined and recorded. Their sympathovagal balance was assessed using the low-frequency-to-high-frequency (LF/HF) ratio of the frequency domain of HRV. Data analysis was performed using SPSS, version 26 (IBM Corp., Armonk, NY). Metric data were represented as numerical values and analyzed as mean ± standard deviation. Student’s t-test was used to compare the LF/HF ratio between the two groups, and Pearson’s correlation coefficient was used to correlate the LF/HF ratio with spirometry data. A *P*-value <0.05 was considered statistically significant.

Results: Cases had a significantly higher LF/HF ratio than controls (1.34 ± 0.45 vs 1.048 ± 0.56; *t* = 3.38, *P* = 0.0007). Among cases, the LF/HF ratio showed a statistically significant inverse correlation with % FEV1 (*r* = −0.772, *P* < 0.0001) and % FVC (*r* = −0.583, *P* < 0.0001). However, its correlation with % FEV1/FVC was not statistically significant (*r* = −0.133, *P* = 0.268). Among controls, the LF/HF ratio showed a statistically significant inverse correlation with % FEV1 (*r* = −0.499, *P* < 0.0001), % FVC (*r* = −0.462, *P* < 0.0001), and % FEV1/FVC (*r* = −0.354, *P* = 0.0023).

Conclusions: Drug-naïve COPD patients have significantly higher sympathovagal dominance in comparison to the apparently healthy control population. Their sympathovagal dominance is inversely correlated with their spirometry parameters. In the control population, sympathovagal dominance is also inversely correlated with spirometry parameters; however, to a lesser extent. This implies that greater sympathovagal dominance is produced to combat an increasing degree of airway obstruction.

## Introduction

Chronic obstructive pulmonary disease (COPD) is a common public health concern in the present era due to decreasing air quality from both outdoor and indoor pollution, as well as cigarette smoking, whether active or passive. Another cause may be genetic, i.e., alpha-1 antitrypsin deficiency. COPD causes inflammatory damage to the bronchoalveolar tree and progressive airway obstruction, which worsens and manifests as chronic bronchitis or emphysema, depending on their variable pathological presentations, both of which are classified under the umbrella of COPD. It has a male preponderance in terms of both occurrence and severity. Patients primarily present with progressive chronic cough, copious sputum production, and dyspnea. Investigation of COPD essentially involves digital spirometry, which assesses Forced Expiratory Volume in the first second (FEV1), Forced Vital Capacity (FVC), and the FEV1/FVC ratio, both before and after inhalation of a short-acting β2 agonist (SABA) bronchodilator [1]. The Global Initiative for Chronic Obstructive Lung Disease (GOLD) provides widely accepted criteria for the diagnosis of COPD severity and thus guides therapy [2].

The sympathetic nervous discharge is initiated as a response to various stressors in the body. Sympathetic β2- receptors are present in the bronchoalveolar tree and mediate bronchodilation upon sympathetic drive activation. In healthy individuals, there is a vagal/parasympathetic dominance throughout the body, essential for physiological balance at rest [3].

The heart rate variability (HRV) is defined as the beat-to-beat variability analysis of the RR interval of ECG signals. It includes time-domain and frequency-domain analyses of ECG signal data, encompassing various parameters. The low-frequency (LF) parameter of the frequency domain of HRV represents both sympathetic drive and, to a lesser extent, parasympathetic activity, whereas the high-frequency (HF) parameter represents purely parasympathetic vagal tone. Various factors are known to affect HRV, including age, sex, cardiovascular disorders, mental comorbidities, and endocrine and metabolic disorders [4]. The low-frequency-to-high-frequency (LF/HF) ratio analysis is, therefore, a relative neurophysiological marker for assessing sympathovagal balance in an individual [5,6].

Previous studies have shown that COPD patients have higher sympathetic excitation and sympathovagal imbalance [6,7,8]. A few studies have also suggested dysregulated vagal tone and decreased HRV in COPD [6,9,10]. However, to the best of our knowledge, the autonomic status of drug-naïve COPD patients, in comparison to healthy subjects, remains unclear. The rationale of our study is to explore the autonomic pathophysiology of drug-naïve COPD patients and how it differs from the physiology of healthy subjects.

Our study aims to compare the HRV (via LF/HF ratio) of drug-naïve COPD patients and age- and sex-matched healthy controls and examine its correlation with spirometry parameters.

We hypothesize that newly diagnosed, drug-naïve COPD patients must have a physiologically disturbed sympathovagal balance to maintain airway patency for sustaining respiration.

## Materials and methods

**Figure 1:**
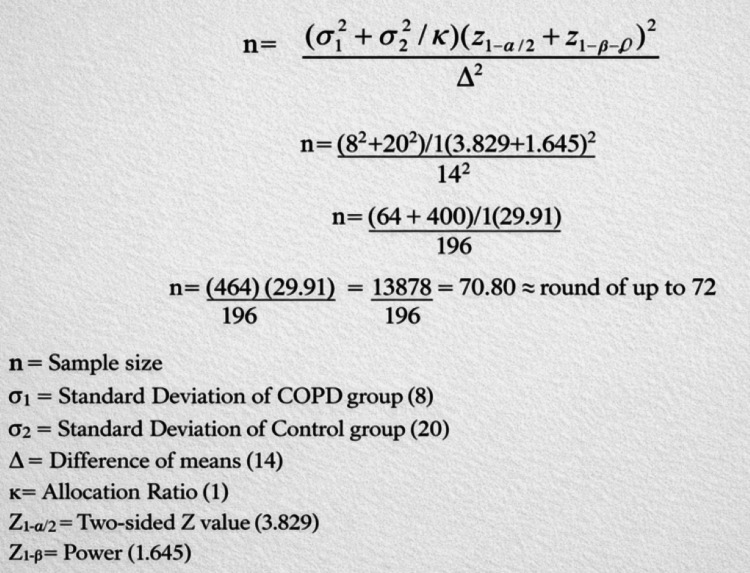
Sample size derivation summary. Source: [12].

Our study was conducted as a cross-sectional analytical study in the Respiratory Medicine outpatient wing of King George’s Medical University (KGMU), Lucknow, India. The study commenced after obtaining ethical clearance from the KGMU Institutional Ethics Committee (reference code: IV PGTSC-IIA/P27). A sample size of 72 was deduced based on a similar study by Stein et al. [11]. We used their root mean square of successive differences (RMSSD), a time-domain parameter of HRV, for sample size calculation. They had compared HRV data of 18 COPD patients and 18 healthy subjects. They obtained a mean difference in RMSSD of 14 ms, with a standard deviation of 8 ms for the COPD group and 20 ms for the control group. The confidence interval (two-sided) was assumed to be 99.99%, and the power was assumed to be 95%. The allocation ratio was kept at 1. This yielded a sample size of 70.8 per group, which we rounded off to 72 subjects per group. The sample size derivation is summarized in Figure 1 (Table 1).

**Table 1:** Summary of sample size derivation. Confidence interval (two-sided)= 99.99%. Power = 95%. Total sample size = 72 in each group. Source: [11].

	24-hour indices rMSSD (ms)		
	COPD group	Control group	Difference*
Mean	31	45	-14
Standard deviation	8	20	
Variance	64	400	

Thereafter, 72 drug-naïve, newly diagnosed COPD patients (on the basis of GOLD criteria) [2] were enrolled as cases in the study, and 72 apparently healthy candidates were selected as controls (Table 2).

**Table 2:** GOLD diagnostic criteria for COPD. An FEV1/FVC ratio of <70% of the predicted value after bronchodilator inhalation. Source: [2]. COPD, chronic obstructive pulmonary disease; FEV1/FVC, forced expiratory volume in the first second-to-forced vital capacity

FEV1 (% predicted)	Severity	Grade
≥80%	Mild	Grade 1
50%-79%	Moderate	Grade 2
30%-49%	Severe	Grade 3
<30%	Very severe	Grade 4

All subjects were selected from the middle-aged group and above (>30 years). Both male and female subjects were enrolled in the study, as available in the Outpatient Department (OPD). However, due to the higher occurrence of COPD in males, the majority of the selected cases were male (54 subjects out of 72). To eliminate gender selection bias, we enrolled the same proportion of subjects in the control group (54 males and 18 females).

A simple convenience-based sampling method was used for COPD cases who came for their first visit to the Respiratory Medicine OPD. In contrast, individualized sampling was performed for controls, who were selected to match various variables such as age, sex, tobacco use, and BMI with the corresponding cases. Informed consent was obtained from all subjects after explaining the procedures of the investigations in detail and the purpose of the study.

Both HRV and spirometry were conducted on the same day for each subject to eliminate any physiological bias. The complete study was conducted over a period of one year (August 20, 2021, to August 20, 2022).

Candidates were excluded if they had pulmonary comorbidities (such as interstitial lung disease, pulmonary tuberculosis, bronchiectasis, or pleural effusion), any known cardiovascular disease or history of autonomic dysfunction, endocrinopathies such as diabetes mellitus and metabolic syndrome, tobacco chewing or alcohol addiction, or other mental illnesses.

Both cases and controls were matched for smoking, lifestyle, and dietary habits through an extensive clinical history. The procedures for clinical tests and evaluations were explained, and written consent was obtained from all candidates.

Anthropometric measurements, along with resting heart rate and blood pressure, were recorded of both cases and controls. Candidates were instructed not to consume tea/coffee for at least six hours before evaluation. Spirometry was performed using ndd Medical Technologies’ Easy on-PC spirometer. Candidates were evaluated in a sitting posture, and their resting and post-bronchodilator spirometry values were recorded.

The HRV analysis was performed using LabChart v8.1.10 (AD Instruments, PowerLab 26T, Sydney, Australia). This test is widely used to evaluate the autonomic status of an individual. The LF parameter is a marker of predominantly sympathetic drive with a mixed influence of parasympathetic tone under resting slow-paced breathing conditions, and the LF/HF ratio serves as a relative marker for assessing sympathovagal balance [5,6]. After a resting phase of supine lying with eyes closed for 15 minutes, a 5-minute epoch of HRV was recorded for each candidate using a slow-paced breathing pattern. Lead calibration was performed in default human mode with the maximum frequency filter set at 0.5 Hz, and ectopic beats were eliminated from the HRV epoch recording. The LF/HF ratio was then analyzed.

## Results

The nominal and numerical data obtained from the subjects were compiled using MS Excel 2019 software. The data were analyzed using SPSS, version 26 (IBM Corp., Armonk, NY). Metric data were analyzed as mean ± standard deviation. Student’s t-test was used to compare the LF/HF ratio between the two groups, and Pearson’s correlation coefficient was used to assess the correlation of the LF/HF ratio with spirometry data. *P* < 0.05 was considered statistically significant.

The age group of both cases and controls was 30-60 years. In both groups, 54 candidates were male and 18 were female. Anthropometric data did not vary significantly between the two groups. All candidates belonged to Southeast Asian ethnicity. Among the 54 men in each group, 42 were smokers. None of the women in either group had a history of active smoking.

**Figure 2:**
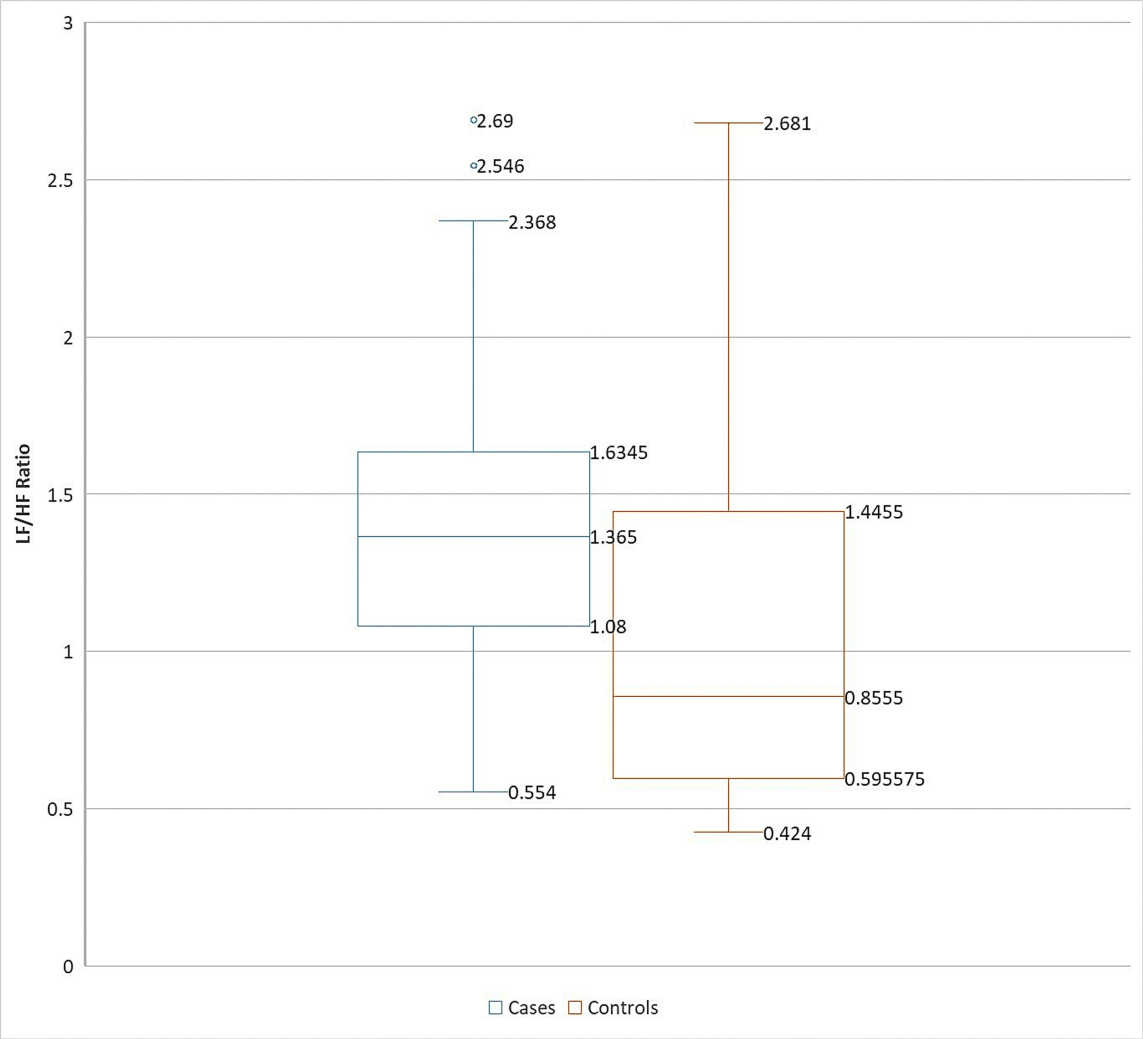
Box-and-whisker plot comparing LF/HF ratios of cases and controls. LF/HF, low-frequency-to-high-frequency

**Figure 3:**
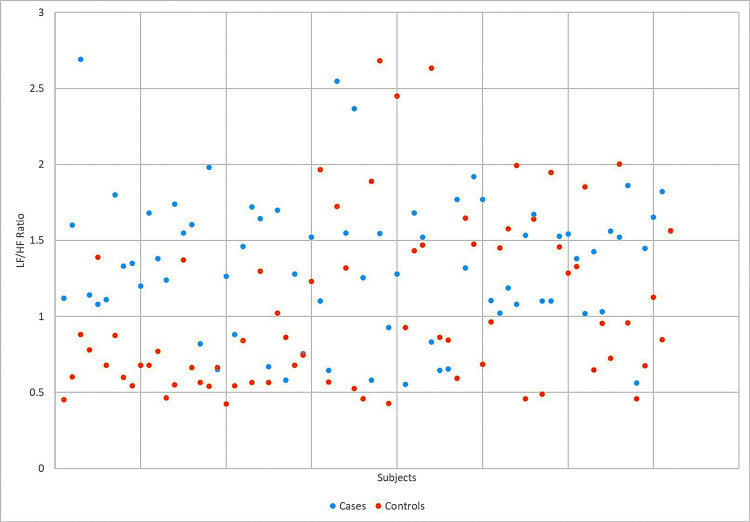
Scatter plot comparing LF/HF ratios of cases and controls LF/HF, low-frequency-to-high-frequency

The drug-naïve COPD cases had a significantly higher LF/HF ratio compared to controls (1.34 ± 0.45 vs. 1.048 ± 0.56; *t* = 3.45, *P* = 0.0007). Their comparative data are represented in the box-and-whisker plot and scatter plot images (Figures 2-3).

**Figure 4:**
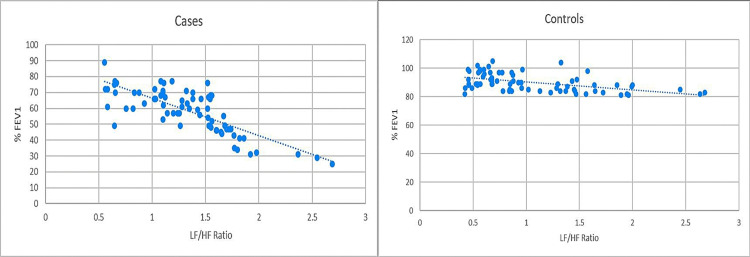
Comparison of correlation between % FEV1 and LF/HF ratio in cases and controls. Pearson’s correlation coefficient for cases (*r* = −0.772, *P* < 0.0001) versus controls (*r* = −0.499, *P* < 0.0001). % FEV1, percentage of expected forced expiratory volume in the first second; LF/HF, low-frequency-to-high-frequency

**Figure 5:**
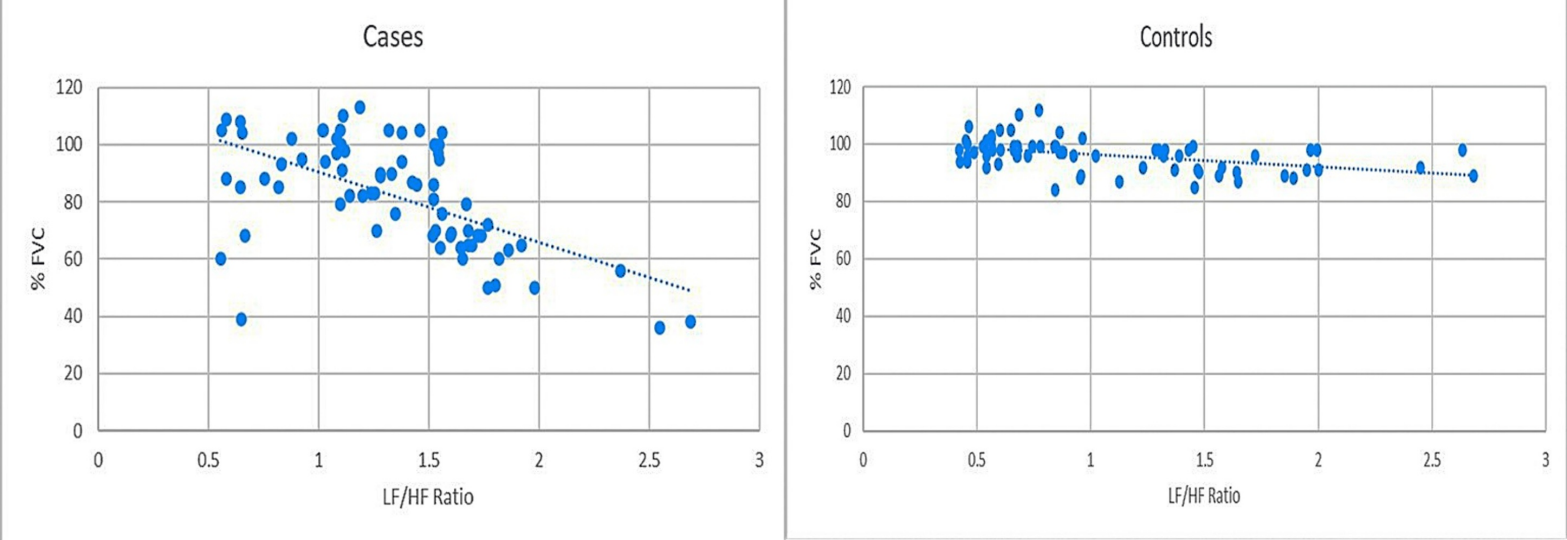
Comparison of correlation between % FVC and LF/HF ratio in cases and controls. Pearson’s correlation coefficient for cases (*r* = −0.583, *P* < 0.0001) versus controls (*r* = −0.462, *P* < 0.0001). % FVC, percentage of expected forced vital capacity; LF/HF, low-frequency-to-high-frequency

**Figure 6:**
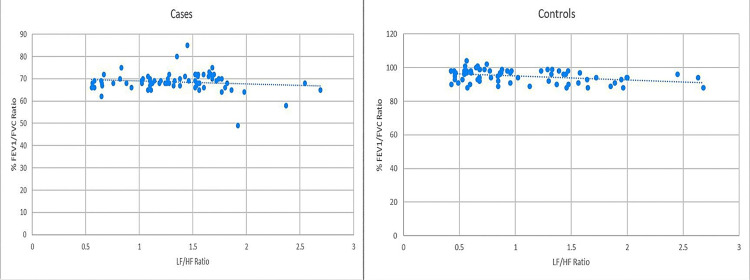
Comparison of correlation between % FEV1/FVC and LF/HF ratio in cases and controls. Pearson’s correlation coefficient for cases (*r* = −0.133, *P* = 0.268) versus controls (*r* = −0.354, *P* = 0.0023). % FEV1/FVC, percentage of expected forced expiratory volume in the first second-to-forced vital capacity ratio; LF/HF, low-frequency-to-high-frequency

Among cases, the LF/HF ratio showed a strongly significant inverse correlation with % FEV1 (*r* = −0.772, *P* < 0.0001) and % FVC (*r* = −0.583, *P* < 0.0001). However, no statistically significant correlation was observed between the LF/HF ratio and % FEV1/FVC (r = −0.133, P = 0.268). Among controls, the LF/HF ratio showed a statistically significant inverse correlation with all three spirometry parameters: % FEV1 (*r* = −0.499, *P* < 0.0001), % FVC (*r* = −0.462, *P* < 0.0001), and % FEV1/FVC (*r* = −0.354, *P* = 0.0023). These correlations are illustrated in Figures 4-6.

## Discussion

The sympathetic nervous system is known to help the body cope with stress and regulates various involuntary activities, including increased heart rate, increased cardiac output, decreased gastrointestinal function, urinary retention, and respiratory bronchodilation [13,14].

Our study found a profound increase in sympathetic drive in COPD patients compared to apparently healthy age- and sex-matched individuals. A plausible explanation for this phenomenon is that, in drug-naïve COPD patients, the activation of presympathetic rostro-ventro-lateral medulla neurons due to central hypoxia may represent a physiological stress response to cope with pathologically constricted bronchial airways [15]. Sympathetic stimulation leads to noradrenergic activation of β2 receptors on bronchial smooth muscles, causing bronchodilation, which can help maintain airflow patency in the bronchial tree of COPD patients [16]. This is supported by similar findings from Spiesshoefer et al. [7], van Gestel and Steier [8], and Heindl et al. [17], who reported a high sympathetic drive in COPD patients.

Jeena et al. [6] also demonstrated a severity-wise increase in sympathetic activity, shown by an increase in the LF parameter and LF/HF ratio in COPD patients, which coincided with the findings of Raju et al. [18] and Chhabra et al. [19]. They also reported a decrease in overall HRV parameters and diminished parasympathetic activity. Bédard et al. [20] conducted a similar study and found that a high LF/HF ratio in COPD patients is independent of anticholinergic and β2-agonist medications. However, contrary to our findings, Camillo et al. [21] concluded that autonomic function in COPD patients is not related to disease severity but mainly depends on the level of daily physical activity.

Our study demonstrated a statistically significant inverse correlation between the LF/HF ratio and % FEV1 and % FVC, and a non-significant inverse correlation with the % FEV1/FVC ratio in COPD patients. We also observed a significant inverse correlation between the LF/HF ratio and % FEV1, % FVC, and % FEV1/FVC ratio in the apparently healthy population. This phenomenon can be explained by the fact that a fall in % FEV1 and % FEV1/FVC ratio is caused by obstructive pathology [22], which may lead to sympathetic stimulation, causing bronchodilation and helping maintain airway patency. A decline in % FVC reflects a reduction in lung capacity to accommodate air [23], and sympathetic stimulation can widen the airway to maintain a required minimum of respiratory function in such a scenario. This rise in sympathetic drive is consistent with a rise in the LF/HF ratio of HRV [4][6]. These findings are consistent with a similar study by Behera et al. [24], who found a weak negative correlation between the LF/HF ratio and various spirometry parameters, which was, however, not statistically significant. Bianchim et al. [25] also demonstrated a weak yet significant correlation between the LF/HF ratio and % FEV1 and % FVC in an apparently healthy population. The cause of the discrepancy between patients and controls may be inherent to the pathophysiology of COPD, which affects FEV1 more than FVC, making the ratio less predictable in patients [26]. To our knowledge, no other study has explored the correlation between the LF/HF ratio and these spirometry parameters.

To date, the sympathovagal response in drug-naïve COPD patients is not well understood, and our study aims to demonstrate an altered sympathovagal balance. This helps expand our understanding of respiratory physiology and its remarkable adaptive capacity in pathological conditions [27].

The limitation of our study is that muscle sympathetic nerve activity (MSNA, a significant marker of sympathetic nervous activity assessed via micro-neurography) [28] could not be incorporated in our study due lack of resources. Another limitation is that other HRV parameters, such as RMSSD, SDRR, LFnu, and HFnu, were not included. Future studies could incorporate these markers to strengthen the evidence for sympathetic drive involvement in the adaptive pathophysiological mechanisms of COPD.

## Conclusions

Based on the results of our study, we deduce that drug-naïve COPD patients cope with respiratory deficiency through a disturbed sympathovagal balance and a relatively increased sympathetic drive, as demonstrated by a higher LF/HF ratio compared to healthy subjects. The severity of sympathovagal discordance is strongly and inversely correlated with spirometry parameters, % FEV1 and % FVC. In comparison, in apparently healthy individuals, sympathetic drive is also inversely correlated with % FEV1 and % FVC, although to a lesser degree, and additionally with the % FEV1/FVC ratio. This can be attributed to normal physiological variation in these individuals and the fact that the majority of controls were smokers (42 out of 72). This study provides clinicians with insights into the natural course of chronic obstructive lung pathology in drug-naïve patients. It also paves the way for future trials and research to investigate the role of sympathovagal imbalance in COPD and to prevent or delay the cardio-pulmonary complications associated with this disturbance.
